# Demographic analysis of patients requiring ophthalmology consultation in the emergency department: A retrospective study at a tertiary care center

**DOI:** 10.1097/MD.0000000000041696

**Published:** 2025-03-07

**Authors:** Yasemin Ozkaya, Emine Kaya-Guner, Duygu Inci-Bozbiyik, Yasemin Kilic Ozturk

**Affiliations:** a Department of Family Medicine, Izmir City Hospital, Izmir, Turkey; b Department of Ophthalmology, Izmir City Hospital, Izmir, Turkey; c Department of Family Medicine, University of Health Sciences Turkey, Izmir Faculty of Medicine Tepecik Training and Research Hospital, Izmir, Turkey.

**Keywords:** consultation, emergency department, eye health, family medicine, occupational health and safety, primary care

## Abstract

This study aims to determine the necessary preventive measures from the perspective of primary care by examining the demographic characteristics and initial diagnoses of patients who were evaluated by an ophthalmologist following an emergency consultation request. Individuals over the age of 18 who had complete data and were referred for ophthalmic consultation from the emergency department within in 2022 were included in the study. The study retrospectively reviewed the patients’ age, gender, diagnoses, status of ophthalmic consultation, whether a forensic report was prepared, and the outcomes of follow-up, hospitalization, or discharge. When examining the diagnoses of patients who were referred for ophthalmic consultation, the majority (60.1%) had eye or periocular trauma, followed by conjunctival diseases (19.8%). Many patients who present to the emergency department with eye complaints have conditions that could be diagnosed in primary care and treated or referred to an eye specialist to prevent further progression. Family physicians serve as the first point of contact for individuals seeking assistance with health issues, highlighting their critical role in protecting eye health. Family physicians should also educate individuals on occupational health and safety, including the prevention and monitoring of occupational accidents and diseases, which make up a significant portion of eye injuries.

## 1. Introduction

Eye injuries are a common occurrence worldwide and are one of the leading causes of preventable blindness, as well as one of the most frequent reasons for visits to ophthalmology emergency departments.^[1]^

The physical and psychological impacts of eye injuries on individuals often result in both direct and indirect costs, such as job loss, significant lifestyle changes, and permanent visual and physical disabilities, placing a substantial burden on families and healthcare systems.^[2]^ It is estimated that 90% of eye injuries are preventable.^[3]^ Therefore, programs aimed at preventing eye injuries are considered critical public health measures.^[2]^

In Turkey, ophthalmologists provide secondary and tertiary healthcare services. However, some specialists accept patients in private clinics or hospitals. Eye health services in primary care are provided by family physicians and are generally limited to basic examinations and referrals. The lack of necessary medical equipment for eye examinations in primary healthcare centers often requires patients to seek direct consultation with ophthalmologists. However, fundamental screening methods and the management of certain eye diseases are feasible within primary care, and these services are available. In this context, determining the number of patients who can be treated in primary care settings or by family physicians instead of emergency departments is of great importance. This approach could help reduce overcrowding in hospital emergency services and enable a more cost-effective healthcare system.

Primary healthcare services are provided by clinical experts who establish close relationships with patients, identify their individual healthcare needs, and assume responsibility within the context of family and community.^[4]^ The initial visit to a primary care physician provides a more cost-effective service delivery for patients and healthcare systems, facilitating timely and easier access to specialists when necessary and maximizing the benefits of primary healthcare services^.[5]^ Occupational eye injuries are common occurrences, and they can largely be prevented through the proper use of personal protective equipment, occupational safety training, and regular eye examinations.^[3]^ Family physicians can raise awareness about eye health by providing patient education, informative brochures, health literacy programs, and personalized consultations.^[6]^ Additionally, organizing training sessions on occupational health and safety for employees and involving family physicians in these processes could be an effective strategy to prevent occupational diseases and eye trauma.

This study aims to determine the necessary preventive measures from a primary care perspective by analyzing the demographic characteristics and initial diagnoses of patients evaluated by an ophthalmologist upon an emergency consultation request.

## 2. Materials and methods

The study commenced following the approval of the Clinical Research Ethics Committee on July 13, 2023, with decision number 2023/06-22. A total of 5626 patients, who presented to the adult emergency department and were referred for ophthalmic consultation between January 01, 2022 and December 31, 2022, and were evaluated by an ophthalmologist, were included in the study. Individuals under the age of 18 or those with incomplete data were excluded from the research.

The study retrospectively reviewed patients’ age, gender, diagnoses, status of ophthalmic consultation, the preparation of forensic reports, and the outcomes of follow-up, hospitalization, or discharge.

Statistical analysis: descriptive statistics for the data included mean, standard deviation, median, minimum, maximum, frequency, and percentage values. Statistical analyses were performed using the SPSS 23.0 software program.

## 3. Results

In 2022, ophthalmic consultations were requested for 5626 individuals who presented to the emergency department. The mean age of the 5626 patients included in the study was 43.2 ± 15.1 years, ranging from 18 to 96 years. When examining the gender distribution of the patients, 76.4% were male and 23.6% were female.

Regarding the diagnoses of patients who were referred for ophthalmic consultation, 60.1% (3383 patients) had eye or periocular trauma, while 19.8% (1116 patients) had conjunctival diseases, followed by eyelid disorders, retinal diseases, and corneal diseases in descending order of frequency.

Based on the frequency of ophthalmic consultations, 2044 patients (36.3%) were diagnosed with “foreign body in the cornea,” 914 patients (16.2%) had “corneal abrasion and corneal injury without foreign body,” and 769 patients (13.6%) were diagnosed with “infectious conjunctivitis” (Table 1).

**Table 1:** Distribution of diagnoses for patients referred for ophthalmic consultation from the emergency department.

	Diagnosis	Number of patients (n = 5626)	%
Trauma to the eye or periocular area (n = 3383)	Foreign body in the cornea	2044	36.3
	Corneal abrasion and conjunctival injury without foreign body	914	16.2
	Other trauma to the eye and periocular area ○ Fracture of skull and facial bones • Fracture of the malar and maxillary bones • Fracture of the orbital floor	166 35 3 7	2.95
	Foreign body in the conjunctival sac	154	2.73
	Ocular laceration and rupture with tissue loss or prolapse	96	1.70
	Hyphema	9	0.15
Conjunctival diseases (n = 1116)	Infectious conjunctivitis	769	13.6
	Non-infectious conjunctivitis	235	4.17
	Conjunctival hemorrhage	107	1.90
	Conjunctival degeneration and deposits	5	0.08
Eyelid diseases (n = 184)	Blepharitis	107	19
	Chalazion	39	0.69
	Herpes with eyelid involvement	20	0.35
	Stye and other deep inflammations of the eyelid	10	0.17
	Bell’s palsy	3	0.05
	Entropion and trichiasis of the eyelid	2	0.03
	Ectropion of the eyelid	1	0.01
	Corrosions of the eyelid and periocular area	1	0.01
	Ptosis	1	0.01
Retinal diseases (n = 104)	Macular and posterior pole degeneration	79	1.40
	Retinal detachment	12	0.21
	Diabetic retinopathy	8	0.14
	Retinal tear without detachment	2	0.03
	Vitreous hemorrhage	2	0.03
	Retinal vascular occlusion	1	0.01
Corneal diseases (n = 46)	Keratitis	33	0.58
	Pterygium	5	0.08
	Corneal dystrophy	4	0.07
	Corneal scars and opacities	4	0.07
Lacrimal system diseases (n = 41)	Disorders of the lacrimal gland	39	0.69
	Dacryocystitis	3	0.05
Glaucoma (n = 39)	Primary open-angle Glaucoma	19	0.33
	Closed-angle glaucoma	20	0.35
Cellulitis (n = 38)	Periorbital cellulitis	37	0.65
	Orbital cellulitis	1	0.01
Uveal and choroidal diseases (n = 32)	Iridocyclitis	27	0.47
	Scleritis	4	0.07
	Episcleritis	1	0.01
Neuro-ophthalmological disorders (n = 15)	Optic neuritis	6	0.10
	Optic nerve disorders	6	0.10
	Papilledema	2	0.03
	Sixth (abducens) nerve palsy	1	0.01
Cataract		17	0.30
Visual impairment and loss (patients with previous permanent vision loss from other causes)		15	0.26
Other refractive disorders		6	0.10
Malignant neoplasm of the eye		2	0.03
No pathology found		587	10.4

Among the patients referred for ophthalmic consultation, forensic reports were prepared for 567 patients (10.1%). Upon examining these reports, it was found that 163 patients (28.7%) were diagnosed with a “foreign body in the cornea,” 127 patients (22.4%) had “corneal abrasion and conjunctival injury without a foreign body,” and 14 patients (2.4%) had a “foreign body in the conjunctival sac.” Other patients with forensic reports were seen in the emergency department due to traffic accidents, falls, assaults, or burns, for which ophthalmic consultation was requested to check for eye trauma.

In this study, the conditions listed in Table 1, including trauma to the eye and periocular area (such as foreign body in the cornea, corneal abrasion, and conjunctival injury without foreign body), herpes with eyelid involvement, Bell’s palsy, corrosive injury, acute ptosis, retinal detachment, tear and retinal vascular occlusions, keratitis, dacryocystitis, glaucoma, periorbital and orbital cellulitis, uveal diseases, and neuro-ophthalmological conditions, were classified as ophthalmic emergencies. In total, 3583 patients were considered to have ophthalmic emergencies, representing 63.6% of all patients.

Among the 587 patients (10.4%) referred for ophthalmic consultation from the emergency department for reasons such as neurology, infection, or trauma, no pathological findings were observed.

When looking at the hospitalization status of the patients after presenting to the emergency department, only 71 of the 5626 patients (1.3%) were admitted to the ophthalmology department, and a total of 176 patients (3.1%) were hospitalized in the hospital.

The average time for the requested ophthalmic consultations was found to be 38.8 ± 65.1 minutes (minimum 1 minute, maximum 31 hours).

## 4. Discussion

According to the findings of the study, the majority of patients referred for ophthalmic consultation from the emergency department presented with trauma to the eye and/or periocular area. Conjunctival diseases were observed as the second most common cause. It was noted that patients presenting with eye and/or periocular trauma generally did not require hospitalization. These results indicate that most eye traumas frequently encountered in emergency departments are treatable.

Primary healthcare services play a crucial role in the diagnosis, treatment, and prevention of many common eye diseases in the community, as well as in the maintenance of eye health. We emphasize that family physicians have a critical role in areas such as screening, preventive healthcare services, trauma and infection prevention, early diagnosis, consultation for emergency cases, and the management of chronic diseases, all of which are essential for protecting and promoting health. In this context, enhancing the knowledge level of primary healthcare services and ensuring the implementation of appropriate interventions in this field will positively impact the eye health of the community.

In our study, the majority of patients referred for ophthalmic consultation from the emergency department were male. This result is consistent with data from other multicenter studies.^[7–9]^ These observations may be associated with the fact that men are more exposed to environmental factors and have a higher risk of infectious diseases and trauma due to the types of work they do.

The average age of patients in our study was 43.2 ± 15.1 years, ranging from 18 to 96 years. Findings consistent with the epidemiological profile of the studied population were obtained, similar to the results of a study conducted by Babineau and Sanchez.^[10]^ Their research revealed that men have a higher prevalence in ophthalmologic emergency departments (52.5%) and that a greater number of visits occurred among young adults aged 20 to 40 years (42.5%).

When examining the diagnoses of patients referred for ophthalmic consultation, it was found that 60.1% (3383 patients) had trauma to the eye or periocular area, while 19.8% (1116 patients) had conjunctival diseases. This was followed by eyelid diseases, retinal diseases, and corneal diseases, in order of frequency. This situation can be explained by the fact that our hospital is a tertiary healthcare facility and is located near industrial areas, which also accounts for the predominance of male patients in our study. Similarly, a study conducted by Pierre Filho et al^[11]^ in Northeast Brazil showed that eye trauma is the primary reason for emergency care. Another study also indicated that trauma is the most common reason for eye-related visits to emergency departments.^[12]^ Providing patients with information about protective measures is crucial for reducing eye injuries. Individuals at risk should be guided on the use of eye protective equipment and informed about the risks of eye injuries and prevention methods. Education on situations to be aware of in daily life, preventive measures that can be taken at the workplace, and safety tips can help in the prevention of eye injuries. In this regard, family physicians play an essential role, as they frequently interact with their patients and can contribute significantly to reducing such injuries.

According to the frequency of ophthalmic consultations requested, 2044 patients (36.3%) were diagnosed with “foreign body in the cornea,” 914 patients (16.2%) with “corneal abrasion and corneal injury without a foreign body,” and 769 patients (13.6%) with “infectious conjunctivitis.” Luo and Gardiner^[13]^ reported similar results, highlighting the high incidence of foreign bodies in the eye and corneal abrasions without foreign bodies. This situation may be related to the types of work these patients engage in and their exposure to trauma. Globally, at least 3 workers are injured every second, and 1 worker dies every 3 minutes due to work accidents or occupational diseases. It should be remembered that occupational health and safety concerns not only the individual but also their family and society.^[14]^ Family health centers play a crucial role in this regard, serving as primary points of contact for individuals seeking help with health issues. They are essential for monitoring and preventing work accidents and occupational diseases, contributing significantly to occupational health and safety. Additionally, consistent with our findings, conjunctivitis is reported in the literature as the most common infectious eye disease.^[15]^ This can be explained by the high contagiousness of the pathology and the lack of proper hygiene.

Upon evaluating the status of patients classified as eye emergencies, it was found that 63.6% of the 5626 patients required urgent examination by an eye specialist. A total of 1265 patients diagnosed with conjunctival diseases, excluding “foreign body in the conjunctival sac” and “conjunctival degeneration and deposits” as listed in Table 1, could have been managed by a family physician or an emergency physician. It was concluded that 22.4% of all patients could have been treated at the primary care level or in the emergency department without the need for ophthalmic consultation. Educating patients on this matter and enhancing the ophthalmological competence of family physicians and emergency physicians could potentially reduce the patient load in tertiary care hospitals.

Among the patients who were referred for an eye consultation, 567 (10.1%) were issued forensic reports. Upon reviewing these reports, it was found that 163 patients (28.7%) were diagnosed with “foreign body in the cornea,” 127 patients (22.4%) with “corneal abrasion and conjunctival injury without a foreign body,” and 14 patients (2.4%) with “foreign body in the conjunctival sac.” The other patients who were issued forensic reports had presented to the emergency department due to incidents such as traffic accidents, falls, assaults, or burns, and eye consultations were requested for trauma assessment. The fact that most of the forensic reports were related to work accidents underscores once again the importance of occupational health and safety.

When examining the hospitalization status of patients after presenting to the emergency department, it was found that only 71 out of 5626 patients (1.3%) were admitted to the ophthalmology department. In total, 176 patients (3.1%) were hospitalized. In a study involving Gülen et al,^[9]^ it was found that a large portion of patients presenting to the emergency department (95.2%) were discharged, with only 4% requiring hospitalization. The significant number of patients being discharged from our emergency department indicates that these individuals could be treated on an outpatient basis and discharged with simple medical interventions.

It is known that accessing primary healthcare services offers a more economical and accessible option for both patients and health systems, but in the absence of a referral system, patients choosing to see specialists on their own can lead to certain issues. Family physicians play a crucial role in guiding patients in this regard. A significant number of patients presenting to the emergency department with eye complaints can have their conditions diagnosed or receive preliminary assessments in primary healthcare settings, enabling them to be referred to eye specialists and potentially preventing disease progression.^[16]^

When evaluating the requested ophthalmology consultation times, it was found that the average consultation duration was 38.8 ± 65.1 minutes (minimum 1 minute, maximum 31 hours). This collaborative process, in which at least 2 doctors evaluate the patient together, is significant in the provision of healthcare services but can sometimes lead to undesirable outcomes. Factors such as rising hospital costs, the complexity of emergency preparedness, and the increase in the number of patients receiving unnecessary services can be considered among the negative impacts of consultations.^[17]^ This situation may lead to longer patient stay durations in emergency departments, increased emergency service congestion, and in some cases, prolonged waiting times that reduce the quality of service.^[18]^ The fact that our hospital provides tertiary healthcare services, the density of patient referrals to the emergency department, and the availability of an on-call ophthalmologist 24 hours a day can be considered reasons affecting the response times for eye consultations from patients with eye complaints. It is believed that diagnosing and referring the majority of commonly encountered eye diseases in the community through primary healthcare services, either by making a diagnosis or by using possible preliminary diagnoses, will help reduce the number of individuals who genuinely need to see an ophthalmologist from making tertiary referrals, thereby contributing to a decrease in consultation times and the limitations created by consultations.

Additionally, the prevention of workplace accidents and occupational diseases is of great importance in reducing the financial burden on workers, their families, and society. The primary responsibility for ensuring a safe working environment lies with employers, who are obligated to implement safety regulations and ensure compliance among employees.^[19]^ Family physicians also play a crucial role in the implementation of preventive measures and patient education regarding occupational health and safety. To support this role, they can collaborate with workplace physicians to organize occupational health and safety training sessions and inform employees. However, it should not be forgotten that the primary preventive power lies within workplaces and health policies.

## 5. Conclusion

According to the results of this study, it has been determined that the majority of patients requesting ophthalmology consultations from the emergency department presented due to eye and/or periorbital trauma. Conjunctival diseases are also another commonly encountered issue.

Our findings suggest that eye injuries and conjunctivitis are frequently situations that require consultation with an ophthalmologist from the emergency department; however, it is believed that educating patients and implementing preventive measures will contribute to reducing patient admissions for these 2 conditions.

The removal of foreign bodies from the eye is an urgent situation that requires immediate intervention, and early action is critical in preventing serious complications. The use of protective equipment against foreign bodies is a fundamental strategy in preventing such accidents, and general awareness and educational programs on this issue are important. Additionally, training family physicians and emergency room doctors in removing foreign bodies from the cornea can help ensure that patients receive accurate and effective treatment. It is crucial that the physicians performing the initial intervention are knowledgeable and competent in the removal of foreign bodies from the eye to improve patient outcomes. All of these measures can contribute to reducing the potential dangers posed by foreign bodies in the eye and safeguarding patients’ health and safety. Educating patients and implementing preventive measures in reducing eye injuries and conjunctivitis cases, along with enhancing community awareness and strengthening the role of family physicians, are of great importance for protection and improving treatment processes.

## Author contributions

**Conceptualization:** Yasemin Ozkaya.

**Formal analysis:** Yasemin Ozkaya.

**Methodology:** Yasemin Ozkaya, Emine Kaya-Guner, Duygu Inci-Bozbiyik, Yasemin Kilic Ozturk.

**Writing – original draft:** Yasemin Ozkaya, Duygu Inci-Bozbiyik.

**Writing – review & editing:** Yasemin Ozkaya, Emine Kaya-Guner, Yasemin Kilic Ozturk.
